# CD51 Intracellular Domain Promotes Cancer Cell Neurotropism through Interacting with Transcription Factor NR4A3 in Colorectal Cancer

**DOI:** 10.3390/cancers15092623

**Published:** 2023-05-05

**Authors:** Tianze Huang, Yanyun Lin, Junguo Chen, Jiancong Hu, Hao Chen, Yanhong Zhang, Bin Zhang, Xiaosheng He

**Affiliations:** 1 Department of Colorectal Surgery, The Sixth Affiliated Hospital, Sun Yat-Sen University, Guangzhou 510655, China; 2 Guangdong Provincial Key Laboratory of Colorectal and Pelvic Floor Diseases, The Sixth Affiliated Hospital, Sun Yat-Sen University, Guangzhou 510655, China

**Keywords:** CD51, colorectal cancer, intracellular domain, perineural invasion

## Abstract

**Simple Summary:**

Perineural invasion is an important cause of postoperative recurrence and metastasis of colorectal cancer, but the mechanisms of perineural invasion are not well characterized. Meanwhile, there is no targeted therapy for perineural invasion in the current treatment of colorectal cancer. In this study, we first show that CD51 can be cleaved by γ-secretase to generate an intracellular domain that promotes perineural invasion. More importantly, we have demonstrated for the first time that pharmacological inhibition of γ-secretase can help prevent perineural invasion in colorectal cancer.

**Abstract:**

The abundant nervous system in intestine provides the basis for perineural invasion (PNI) of colorectal cancer (CRC). PNI is defined as the invasion of the nerves by cancer cells. Although PNI is already known to be an independent prognostic factor in CRC, the molecular mechanism underlying PNI remains obscure. In this study, we first demonstrated that CD51 could promote the neurotropism of tumor cells through cleavage with γ-secretase to generate an intracellular domain (ICD). Mechanistically, ICD of CD51 could bind to the transcription factor NR4A3, and act as a coactivator to promote the expression of downstream effectors, such as NTRK1, NTRK3, and SEMA3E. Pharmacological inhibition of γ-secretase impedes PNI mediated by CD51 in CRC both in vitro and in vivo and may become a potential therapeutic target for PNI in CRC.

## 1. Introduction

Colorectal cancer (CRC) ranks third in terms of incidence and is the third leading cause of cancer-related deaths worldwide, leading to a heavy burden on social economy and public health [1,2]. Numerous risk factors have been reported to be correlated with CRC prognosis, including perineural invasion (PNI), which is identified as the invasion of the nerve by cancer cells. PNI is a common characteristic of some solid tumors [3]. An increasing number of studies have suggested that PNI is involved in tumor progression and recurrence [4,5]. The gastrointestinal tract has evolved with abundant intrinsic nerve connections known as enteric nervous system (ENS) [6]. This rich distribution of nerves may provide favorable conditions for PNI in CRC. The incidence of PNI in CRC ranges from 8% to 42% in different studies [7]. In addition, several previous studies have reported PNI as an independent risk factor associated with worse outcomes in CRC [8,9,10,11]. According to the Society of Clinical Oncology (ASCO) guidelines, perineural invasion is a high-risk factor for adjuvant chemotherapy in stage II colon cancer. However, the benefit of chemotherapy in phase II patients with PNI is not significant [12]. Until now, there has been no targeted treatment for patients with PNI. Although PNI in CRC is gradually attracting increasing attention, our understanding of molecular mechanisms of PNI in CRC remains limited.

CD51, also known as integrin αv, is a member of the integrin family. As type I transmembrane protein [13], CD51 can interact with any of the five β integrin subunits (β1, β3, β5, β6, or β8) to form heterodimers and mediate links between extracellular matrix and cytoskeleton to execute its cellular function [14]. CD51 has been reported to be dysregulated in a variety of tumors and participates in promoting tumor progression by many processes, such as cell adhesion, migration, and signal transduction [15,16,17]. In addition, our previous study demonstrated that CD51 was a functional marker of CRC stem cells [18]. These findings suggest that CD51 plays an important role in CRC. Notably, previous studies have reported a potentially positive association between high CD51 expression and PNI in CRC patients [19,20]. However, the direct relationship between CD51 and PNI has not been thoroughly explored.

γ-Secretase is a well-studied protease belonging to a family of intramembrane proteases, consisting of four essential member proteins: presenilin (PS), nicastrin (NCT), anterior pharynx defective-1 (APH-1), and presenilin enhancer2 (PEN2) [21]. Assembly of these four members is necessary for their proteolytic function. As a protease, γ-secretase contains a wide range of substrates, of which type I transmembrane proteins are the most well studied [22]. Numerous studies have focused on the proteolytic ability of γ-secretase to generate amyloid β-peptide from the amyloid precursor protein, which contributes to the pathogenesis of Alzheimer’s disease [23]. Moreover, γ-secretase may also be involved in regulating the biological behavior of tumors by disturbing various pathways, such as Notch [24] and multiple receptor tyrosine kinases [25,26]. Over the decades, γ-secretase has been considered as a potential therapeutic target for cancer.

Herein, we found that CD51 was upregulated in CRC patients with PNI and associated with poor prognosis. We demonstrated for the first time that CD51 was involved in PNI in CRC. In this process, intramembrane cleavage of CD51 by γ-secretase to produce an intracellular domain (ICD) was a critical step. Mechanistically, the ICD of CD51 could enter the nucleus and act as a coactivator of the transcription factor NR4A3 to regulate downstream gene expression. Importantly, pharmacological inhibition of γ-secretase may be a potential therapeutic strategy for improving the outcome of PNI in CRC patients.

## 2. Materials and Methods

### 2.1. Clinical Samples

All clinical samples were obtained from patients who underwent surgical resection of primary CRC at the Sixth Affiliated Hospital of Sun Yat-Sen University (Guangzhou, China). The diagnosis of CRC was confirmed by at least 2 professional pathologists. None of the included patients had received neoadjuvant radiotherapy or chemotherapy. The study protocol using human specimens was approved by the Ethics Review Committee of the Six Affiliated Hospitals, Sun Yat-sen University. In this study, 116 formalin-fixed, paraffin-embedded CRC samples were used for immunohistochemistry and 4 pairs of fresh frozen tissue samples were used for western blotting.

### 2.2. Cell Lines and Cell Culture

The American Type Culture Collection provided human CRC cell lines SW480, Caco-2, and rat Schwann cell line RSC96 (ATCC, Manassas, VA, USA). All cells were maintained in Dulbecco’s modified Eagle’s medium (DMEM, Invitrogen, Carlsbad, CA, USA) containing 10% fetal bovine serum (FBS, Invitrogen, Carlsbad, CA, USA) and 1% penicillin/streptomycin and cultured at 37 °C in humidified air with 5% CO_2_. All experiments were performed with mycoplasma-free cells.

### 2.3. Lentiviral Production and Cell Transfection

Lentiviral-mediated cell transfection was performed as described previously [27]. Briefly, target plasmids mixed with packaging plasmids (pSPAX2 and pMD2.G) were co-transfected into 293T cells using lipo3000 (Invitrogen, Carlsbad, CA, USA) according to the manufacturer’s protocol. A 0.45 μm filter was used to filter the viral supernatant collected 48 h and 72 h after post-transfection. The filtered viral supernatant was then added to cancer cells. At 48 h after infection, the cells were screened for antibiotics based on the resistance of the plasmid. Western blotting was used to assess the transfection efficiency after screening.

### 2.4. RNA Extraction and Quantitative Polymerase Chain Reaction (qPCR)

Total RNA was extracted using TRIzol reagent (Invitrogen, Carlsbad, CA, USA) according to the manufacturer’s instructions. Total RNA was reverse-transcribed to cDNA using a HiScript III RT SuperMix for qPCR Kit (Vazyme, Nanjing, China). The cDNA was used for qPCR using ChamQ Universal SYBR qPCR Master Mix (Vazyme, China). All qPCR data were calculated and analyzed using the ΔΔCT method. The mRNA expression levels of GAPDH were used as controls for normalization. All the primer sequences used in this study are listed in Table S1.

### 2.5. Protein Extraction and Western Blotting (WB)

Total protein was extracted using RIPA lysis buffer containing a protease inhibitor. Cytoplasmic and nuclear proteins were isolated using a nuclear cytosol extraction kit. Standard WB procedures were performed as described previously [28]. All antibodies used in this study are listed in Table S2.

### 2.6. Immunohistochemistry (IHC)

Formalin-fixed paraffin-embedded tissues were collected as described above. According to standard IHC procedures, all tissues were subjected to antigen retrieval in a sodium citrate repair solution for 15 min. After blocking with goat serum, sections were incubated with the corresponding primary antibody at 4 °C overnight. The secondary antibody was added and a detection system with DAB solution was applied to explore the antigen the next day. Hematoxylin was used to stain nuclei. All slides were estimated and scored by two pathologists separately according to the proportion of tumor cells with positive staining and signal intensity as described previously [18].

### 2.7. Immunofluorescence (IF)

For immunofluorescence analysis, the cells were fixed with 4% paraformaldehyde and permeabilized in 0.3% Triton X-100 for 10 min. After blocking with goat serum, sections were incubated with the corresponding primary antibody at 4 °C overnight. The secondary antibody was added, and the cell nuclei were stained with DAPI before observation. A LSM880 confocal microscope (Zeiss, Pleasanton, CA, USA) was used to collect images at room temperature.

### 2.8. Luciferase Reporter Assay

Luciferase reporter assays were performed using a Dual-Glo Luciferase Assay System (Promega cat. no. E2920) according to the manufacturer’s protocol. Briefly, an NR4A3 regulatory reporter was constructed by inserting the NR4A3 binding motif (AAAGGTCA) into the pGL4-basic vector. The constructed luciferase reporter plasmid was transfected into SW480 and Caco-2 cells that overexpressed CD51-ICD or empty plasmids. The Renilla luciferase plasmid was transfected simultaneously as an internal control. Firefly and Renilla luciferase activities were measured at 48 h after transfection. Each experiment was performed in triplicates.

### 2.9. Transwell Assay

The co-culture migration assay was performed in a 24-well plate using 8-μm-pore Transwell filters (Corning, Corning, New York, USA, 353097) according to the manufacturer’s instructions. Briefly, 1 × 10^5^ cancer cells were seeded in the upper chamber, while DMEM containing 10% FBS or RSC96 cells was added to the lower chamber and then co-cultured for 48 h. After incubation, the transmembrane cells were fixed with 4% paraformaldehyde and observed under a microscope for counting and subsequent analysis.

### 2.10. Time-Lapse Cell Motility Assay

For cell motility assay, human CRC cell lines SW480 and Caco-2 were co-cultured indirectly with RSC96 cells for 48 h using a 0.4-μm-pore chamber (Corning, Leighton Township, MI, USA, 3450). RSC96 cells were seeded in the upper chamber, while cancer cells were added to the lower chamber. After co-culture for 48 h, cancer cells were collected by trypsin digestion and seeded into 24-well plates at 20,000 cells per well. The movement of cancer cells was recorded continuously using a high-content analysis system (PerkinElmer, Altham, MA, USA) for 6 h. The migration speed of the individual cells was determined and analyzed.

### 2.11. Murine Sciatic Nerve Injection and Functional Evaluation

In situ sciatic nerve injections were performed as previously described [29]. Briefly, BALB/c nude mice were anesthetized using isoflurane (5% for induction of anesthesia and 1–3% for maintenance), and their right sciatic nerve was exposed. After exposure, 5 μL of a cell suspension of 1 × 10^5^ cells per microliter was injected into the sciatic nerve using a 10 μL microsyringe. Surgical sutures were used to close the wounds. The sciatic function score and sciatic nerve index were evaluated according to previously described methods [30]. Briefly, the extension length between the first and fifth toes of the hind limbs was used to estimate sciatic nerve function, while limb function was graded according to the response of the hind limb paw to manual extension of the body, from 5 (normal) to 1 (total paw paralysis). All observations were evaluated and recorded once a week until the mice were sacrificed in the 4th week after surgery. When the mice were killed, their sciatic nerves were exposed and photographed to assess the severity of the PNI. All mouse experiments were approved by the Animal Care and Use Committee of Sun Yat-Sen University (Ethics Approval # IACUC-2022030601).

### 2.12. Co-Immunoprecipitation (Co-IP) Assay

The Co-IP assay was performed as previously described [31]. In brief, lysates from HA-CD51-ICD transfected 293T cells were purified by centrifugation, and the supernatants were retained using protein A and G magnetic beads precooled with anti-HA antibody. After immunoprecipitation, the harvests were collected for subsequent mass spectrum analysis, and WB was performed to verify the direct interactions between CD51-ICD and the proteins found in the mass spectrum.

### 2.13. Chromatin Immunoprecipitation (ChIP) Assay

For ChIP assay, a ChIP assay kit (Cell Signal Technology, Danvers, Massachusetts, USA) was used, and a standard procedure was performed according to the manufacturer’s protocol. Briefly, DNA extracted from lytic cancer cells was digested into fragments of 150–900 bp using micrococcal nuclease. An anti-FLAG antibody or protein A and G magnetic beads were added to the lysates and immunoprecipitated overnight. Then, the enriched chromatin was eluted and collected from the antibody/protein A and G magnetic beads for subsequent sequencing or qPCR analysis.

### 2.14. Statistical Analysis

All continuous variables were expressed as mean or median. Student’s *t*-test or Wilcoxon rank-sum test was used for continuous variables according to the respective applicable conditions. We applied Kaplan–Meier survival analysis and log-rank test to evaluate the prognostic value of CD51 according to PNI status among CRC patients. All statistical analyses were performed using SPSS software (version 26.0) or Prism software (version 8.4). All statistical tests were performed on two sides, and a *p*-value < 0.05 was considered statistically significant. Unless otherwise noted, all the results were obtained from at least three independent replicates.

## 3. Results

### 3.1. CD51 Is Upregulated among PNI Patients and Associated with Worse Prognosis

**Figure 1:**
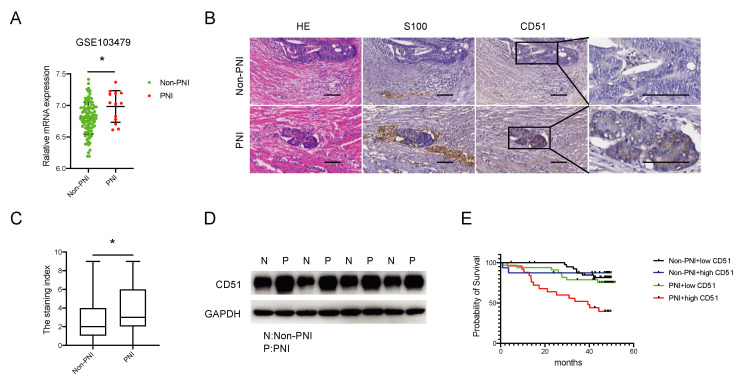
CD51 is upregulated among PNI patients and associated with worse prognosis. (**A**) The expression level of CD51 is higher among perineural invasion (PNI) patients compared to non-PNI patients in the GSE103479 dataset. * *p* < 0.05, two-tailed Student’s *t*-test. (**B**) Representative images of immunohistochemistry (IHC) show that CD51 is upregulated in PNI patient samples compared to non-PNI patient samples. S100 is used as a specific marker to stain nerve fibers. Scale bars, 100 μm. (**C**) Statistical result of immunohistochemistry. The staining index of CD51 is presented. * *p* < 0.05, two-tailed Student’s *t*-test. (**D**) Western blotting (WB) analysis of CD51 protein expression in PNI and non-PNI patients’ samples. (**E**) Kaplan–Meier survival analysis of overall survival (OS) based on patient PNI status and CD51 expression.

In order to determine the difference in CD51 expression between colorectal cancer (CRC) patients with and without perineural invasion (PNI), we analyzed the expression levels of CD51 in the GEO database (GSE103479). The results indicated a higher expression of CD51 in patients with PNI (Figure 1A). To further validate these findings, we conducted differential expression analyses of CD51 using both immunohistochemistry (IHC) and western blotting (WB) on tissue samples from the Sixth Affiliated Hospital of Sun Yat-Sen University. The IHC assay (Figure 1B,C) and WB analysis (Figure 1D) both confirmed that CD51 expression was upregulated in patients with PNI. Furthermore, survival analysis, conducted using the Kaplan–Meier survival method and log-rank test, revealed that CRC patients with PNI and high CD51 expression had worse outcomes. The hazard ratio of “PNI + high CD51” group compared to “Non-PNI + low CD51”, “Non-PNI + high CD51”, and “PNI + low CD51” groups are 6.00 (2.41–14.92), 3.60 (1.37–9.43), and 3.36 (1.44–7.85), respectively (Figure 1E). Overall, these results suggest that CD51 is dysregulated in CRC patients with PNI and is negatively associated with their prognosis.

### 3.2. CD51 Affects the Neurotropism of CRC Cells In Vitro and In Vivo

**Figure 2:**
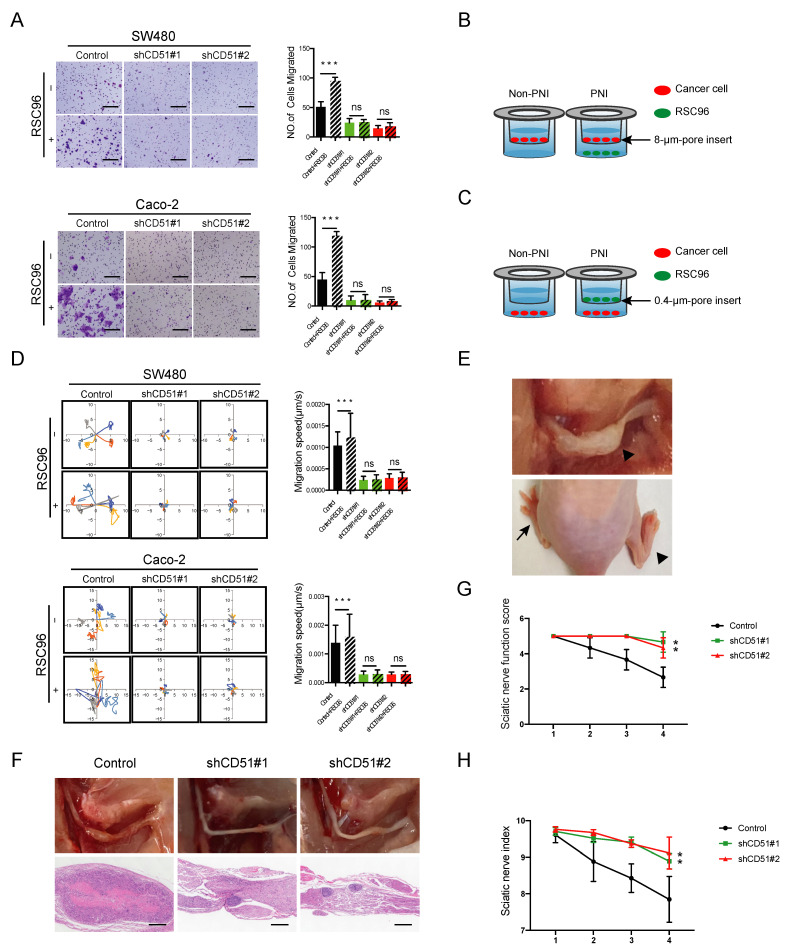
CD51 affects the neurotropism of CRC cells in vitro and in vivo. (**A**) Statistics and representative images of migration of the shControl and shCD51 SW480 and Caco-2 cells. *** *p* < 0.001, two-tailed Student’s *t*-test. Scale bars, 100 μm. (**B**) Schematic of the co-culture migration assay using 8-μm-pore transwell filters. RSC96 cells or only fetal bovine serum (FBS) were seeded in the lower chamber; meanwhile, CRC cells were added into the upper chamber and then co-culture for 48 h. The migration number of CRC cells was then measured. (**C**) Schematic of the co-culture migration speed assay using 0.4-μm-pore transwell filters. RSC96 cells or only fetal bovine serum (FBS) were seeded in the upper chamber; meanwhile, CRC cells were added into the lower chamber and then co-culture for 48 h. The speed of CRC cells was then measured. (**D**) Wind rose plots and statistics of migration speed of the shControl and shCD51 SW480 and Caco-2 cells. Each curve represents a cell trajectory. *** *p* < 0.001, two-tailed Student’s *t*-test. (**E**) The upper shows in situ image of sciatic nerve after injection of SW480 cells. The lower shows representative images of two different functional conditions of the sciatic nerve. The arrow points to the normal hind limb, while the arrowhead marks the functionally impaired hind limb. (**F**) In situ images of sciatic nerve after injection of shControl or shCD51 SW480 cells for 4 weeks and the corresponding H&E-stained images. Scale bars, 100 μm. (**G**,**H**) Statistic of sciatic nerve functions score and sciatic nerve index of mice bearing the shControl or shCD51 SW480 cells for 4 weeks after in situ sciatic injection. ** *p* < 0.01, two-tailed Student’s *t*-test.

To explore whether CD51 could affect PNI, we knocked down endogenous CD51 expression levels in two common CRC cell lines, SW480 and Caco-2, using lentiviral-mediated shRNA interference. Next, using transwell assay (Figure 2B), we observed a significant increase in the migration of cancer cells (upper chamber) co-cultured with rat Schwann cell line RSC96 cells (lower chamber) compared with only fetal bovine serum (FBS) added. Interference of CD51 expression inhibited the migration of cancer cells attracted by RSC96 (Figure 2A). Moreover, we applied time-lapse cell motility assay using a 0.4-μm-pore chamber (Figure 2C) to evaluate cancer cell motility (lower chamber) after stimulation by RSC96 cells (upper chamber). After co-culture, RSC96 cells significantly promote cancer cells’ motility speed. Notably, both SW480 and Caco-2 cells showed impaired mobility in response to RSC96 stimulation following CD51 knockdown (Figure 2D). To determine whether the promotion of PNI by CD51 requires direct cell-to-cell contact, we repeated transwell assay and time-lapse cell motility assay using the supernatant (SN) of RSC96 cells and obtained the same results, which meant the promotion was associated with the cytokines secreted by RSC96 cells (Figure S1A,B). These results indicated that the knockdown of CD51 limited the migration of CRC to RSC96 cells.

To investigate the effect of CD51 knockdown on cancer cell neurotropism in vivo, we used the sciatic nerve injection model described in previous studies [32]. In this murine model, the sciatic nerve function score and sciatic nerve index were used to assess the severity of nerve impairment (Figure 2E). As shown in Figure 2G,H, the sciatic nerve function score and sciatic nerve index in the shCD51 group were less affected than those in the control group, which suggested a role for endogenous CD51 in nerve invasion. The sciatic nerve was exposed after the mice were sacrificed. The ability of cancer cells to invade the sciatic nerve was significantly inhibited by CD51 knockdown. The H&E-stained images further corroborated the findings of the in situ observations (Figure 2F). In summary, our animal model further confirmed that endogenous CD51 promoted the neurotropism of cancer cells in vivo.

### 3.3. CD51 Can Be Cleaved by γ-Secretase to Generate an Intracellular Domain (ICD)

**Figure 3:**
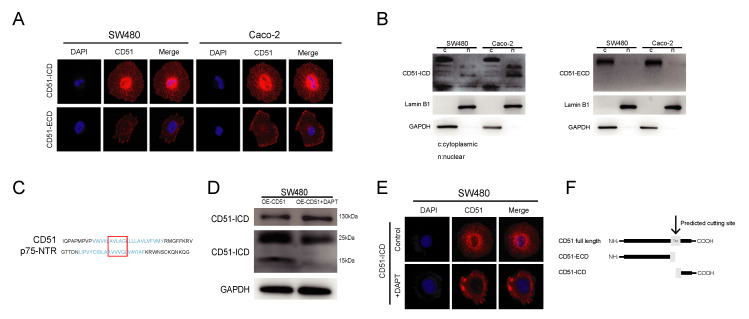
CD51 can be cleaved by γ-secretase to generate an intracellular domain (ICD). (**A**) Representative images of immunofluorescence staining using two different antibodies that recognize the extracellular and intracellular immunogen of CD51, respectively. (**B**) WB analysis of cytoplasmic and nuclear proteins in SW480 and Caco-2 cancer cells using the antibodies that recognize the extracellular and intracellular immunogen of CD51. (**C**) Alignment of CD51 amino acid sequence with amino acid motif sites recognized and cleaved by γ-secretase. The transmembrane region of the protein is highlighted in blue, and the putative similar sequences are circled inside the red box. (**D**) WB images showing changes in protein expression upon addition of the γ-secretase inhibitor DAPT to CD51 overexpressing cells. Protein expression at positions near 15 kd is decreased, the region where the ICD of CD51 is located. (**E**) Representative images of immunofluorescence using CD51-ICD antibody detection after treatment with or without DAPT. (**F**) Schematic of the CD51 transmembrane region being recognized and cleaved by γ-secretase to generate an ICD.

CD51, also known as integrin αv, is a member of the integrin family. As previously reported, integrins regulate downstream functions in a variety of ways, such as conformational changes, protein–protein interactions, and endocytosis, to transmit downstream signals [33]. To explore the mechanism by which CD51 functions, we used the IF assay to investigate the cellular localization of CD51. As shown in Figure 3A, the use of an anti-CD51 intracellular domain (CD51-ICD) antibody marked the expression of CD51 at both the cell membrane and nucleus. We verify antibody specificity by knocking down CD51 and staining it with the ICD antibody. The result showed that the signal intensity significantly decreased after knocking down CD51 (Figure S2A). This suggested that, as a membrane protein, CD51 might also enter the nucleus. Surprisingly, the anti-CD51 extracellular domain (CD51-ECD) antibody only showed binding to antigens on the cell membrane. This phenomenon suggested that CD51 might not enter the cell as a full-length protein but instead was cleaved into different fragments to enter the cell for function. To verify this hypothesis, cytoplasmic and nuclear proteins of SW480 and Caco-2 cells were extracted and detected by WB assay. The results showed that CD51 could be detected in the nucleus using the CD51-ICD antibody, whereas the CD51-ECD antibody failed to detect any specific binding peptide in nuclear-extracted proteins (Figure 3B). This proved that CD51 could be cleaved to generate a small fragment that could enter the nucleus.

As a member of the integrin family, CD51 is a type I transmembrane protein [13]. The intracellular segments of type I transmembrane proteins are mainly produced by the cleavage of γ-secretase. γ-Secretase belongs to a family of intramembrane proteases, which have been reported to be responsible for the release of soluble intramembranous domains during signal transduction [22]. Taken together, we hypothesized that γ-secretase is a candidate for cleavage of CD51 to produce intracellular fragments. To verify this hypothesis, we first searched for the amino acid sequence cleavage site of the substrate of gamma-secretase and found that p75-NTR can be cleaved because it contains an AXXXG motif in the transmembrane domain [34,35]. After comparison, we found that CD51 has the same motif in its transmembrane domain (Figure 3C). Subsequently, we performed IF co-localization of CD51 and the catalytic subunit of γ-secretase, presenilin 1, and found that they co-localize (Figure S2B). Furthermore, we used the γ-secretase inhibitor DAPT to treat cancer cells and investigated the effect of this stimulation on the production of different CD51 fragments. WB assay showed that the expression of CD51 full-length protein increased after DAPT was added. Meanwhile, a reduction was detected near 15 kDa, the region where the short fragment of CD51 was located (Figure 3D). In addition, we treated the cells with DAPT in culture, and then performed IF using an ICD antibody. We found that the nuclear signal weakened after the addition of DAPT, indicating that DAPT inhibited the entry of CD51-ICD into the nucleus (Figure 3E). We also found that co-culturing with RSC96 cells led to an increase in ICD expression but did not lead to an increase in presenilin expression, possibly due to an increase in γ-secretase activity (Figure S2C). In summary, we showed that CD51 could be cleaved by γ-secretase to produce a short ICD and enter the nucleus. A schematic representation of this process is shown in Figure 3F.

### 3.4. Ectopic Overexpression of CD51-ICD Also Promotes PNI in Cancer Cells

**Figure 4:**
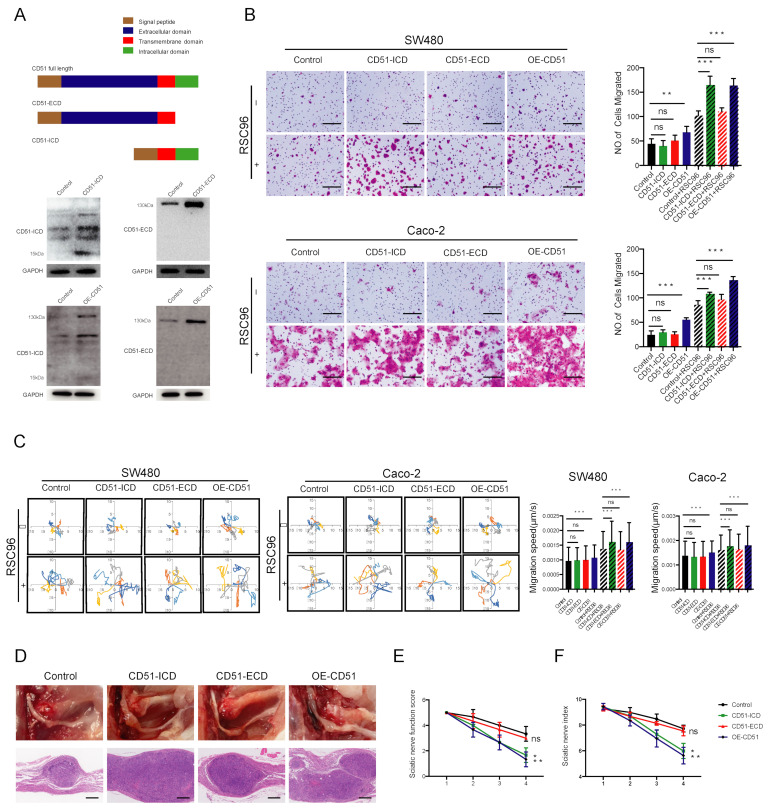
Ectopic overexpression of CD51 ICD also promotes PNI in cancer cells. (**A**) Schematic diagrams for CD51-ICD, CD51-ECD and OE-CD51 vectors, respectively. WB analysis using the antibody which recognizes the CD51 intracellular and extracellular domain immunogen, respectively, to verify the ectopic overexpression in cancer cell lines. (**B**) Statistics and representative images of migration of the SW480 and Caco-2 cells transfected with empty vector, CD51 full-length vector, CD51-ICD vector, and CD51-ECD vector. ** *p* < 0.01, *** *p* < 0.001, two-tailed Student’s *t*-test. Scale bars, 100 μm. (**C**) Wind rose plots and statistical analysis of the migration speeds of the SW480 and Caco-2 cells transfected with empty vector, CD51 full-length vector, CD51-ICD vector, and CD51-ECD vector. Each curve represents a cell trajectory.*** *p* < 0.001, two-tailed Student’s *t*-test. (**D**) In situ images of sciatic nerve after injection for 4 weeks using SW480 cells transfected with empty vector, CD51 full-length vector, CD51-ICD vector, and CD51-ECD vector. In addition, the corresponding H&E-stained images. Scale bars, 100 μm. (**E**,**F**) Statistic of sciatic nerve function score and sciatic nerve index of mice after injection for 4 weeks using SW480 cells transfected with empty vector, CD51 full-length vector, CD51-ICD vector, and CD51-ECD vector. * *p* < 0.05, ** *p* < 0.01, two-tailed Student’s *t*-test.

To investigate the role of different CD51 fragments in promoting neurotropism in CRC cells, CD51 was divided into an N-terminal ECD and a C-terminal ICD, and the corresponding fragment was overexpressed in SW480 and Caco-2 cell lines. We performed WB analyses using antibodies that recognize different terminal immunogens to confirm ectopic overexpression efficiency (Figure 4A). To confirm the efficiency of overexpression, we performed WB detection on cells overexpressing ICD, ECD, and OE-CD51 vectors after knocking down wild-type CD51 (Figure S3A). Notably, the migration ability of cancer cells attracted by RSC96 cells was enhanced only in the CD51 full-length and CD51-ICD overexpression groups, while CD51-ECD was not affected (Figure 4B). In addition, the time-lapse cell motility assay showed similar results (Figure 4C). We repeated transwell assay and time-lapse cell motility assay using the SN of RSC96 cells and obtained the same results (Figure S4A,B). In vivo, the tumor volume in the CD51 full-length and CD51-ICD groups was significantly larger than that in the control group in the in situ and HE-staining section images (Figure 4D). Although the CD51-ECD group also showed slightly increased tumor volume, this group had a much smaller impact than the other two groups. Sciatic nerve function score and sciatic nerve index were evaluated and recorded once a week. As shown in Figure 4E,F, both indices were impaired in the CD51 full-length and CD51-ICD groups, indicating more serious damage to the sciatic nerve. However, the scores of the CD51-ECD group were not significantly different from those of the control group. Combining the results of in vitro and in vivo experiments, we concluded that CD51 might exert its function of promoting the PNI of cancer cells through the CD51-ICD. In addition, WB analysis showed an increase in CD51-ICD expression in patients with PNI (Figure S3B).

### 3.5. CD51-ICD Regulates PNI by Acting as a Coactivator of Transcription Factor NR4A3

**Figure 5:**
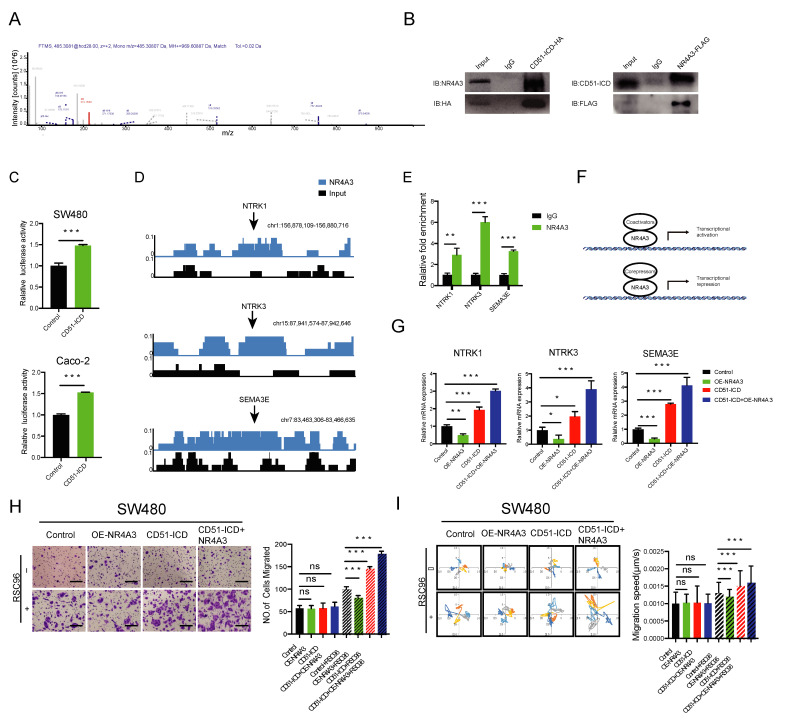
CD51-ICD regulates PNI by acting as a coactivator of transcription factor NR4A3. (**A**) Mass spectrometry analysis shows that CD51-ICD can bind to the transcription factor NR4A3. (**B**) Co-IP and reverse IP of NR4A3 and CD51-ICD.IgG was used as the negative control. (**C**) Statistical analysis of luciferase reporters performed to determine the effect of CD51-ICD on NR4A3 transcriptional activity. *** *p* < 0.001, two-tailed Student’s *t*-test. (**D**) ChIP sequencing analysis revealed the enrichment of NR4A3 in the promoter regions of several PNI-related genes, including NTRK1, NTRK3, and SEMA3E. (**E**) The enrichment region of NR4A3 in promoters of NTRK1, NTRK3, and SEMA3E was verified by ChIP-qPCR. ** *p* < 0.01, *** *p* < 0.001, two-tailed Student’s *t*-test. (**F**) Schematic representation of NR4A3 binding with coactivator or corepressor to regulate downstream gene transcription. (**G**) Statistical analysis of the relative mRNA levels of three PNI-related genes in the corresponding groups transfected with empty vector, NR4A3 vector, CD51-ICD vector, or both. * *p* < 0.05, ** *p* < 0.01, *** *p* < 0.001, two-tailed Student’s *t*-test. (**H**) Statistics and representative images of the migration of cancer cells transfected with corresponding vectors. *** *p* < 0.001, two-tailed Student’s *t*-test. Scale bars, 100 μm. (**I**) Wind rose plots and statistics of the migration speed of cancer cells transfected with corresponding vectors. Each curve represents a cell trajectory. *** *p* < 0.001, two-tailed Student’s *t*-test.

As CD51-ICD could be detected in the nucleus, we suspected that it could directly bind to the chromatin to regulate downstream signaling. However, we failed to detect any specific DNA signal in our chromatin immunoprecipitation (ChIP) assay. Next, we speculated whether CD51-ICD could bind to other proteins to act as an indirect regulatory factor, as other protein ICDs have been reported previously [36]. Therefore, we performed a co-immunoprecipitation (Co-IP) assay and mass spectrometry analysis in 293T cells stably transfected with the CD51-ICD-HA vector. Proteins potentially interacting with CD51-ICD were immunoprecipitated using magnetic beads coated with anti-HA antibody. After excluding non-specific binding proteins from the IgG control, 78 proteins were identified. The proteins that interact with CD51-ICD are listed in Table S3. Among these proteins, we focused on discovering related factors involved in transcriptional regulation and found the only non-general transcription factor, NR4A3 (Figure 5A) [37]. Next, we further validated the interaction using Co-IP and reverse IP assays on CD51-ICD and NR4A3, and the results showed that CD51-ICD and NR4A3 can bind to each other (Figure 5B). To confirm whether the interaction could regulate the transcriptional activity of NR4A3, NR4A3-binding regulatory elements were cloned into the pGL4 luciferase reporter vector and then transfected into CRC cells overexpressing empty vector or ICD vector for luciferase reporter assay. The results showed that CD51-ICD overexpression enhanced the luciferase activity of the NR4A3 reporter (Figure 5C). Collectively, CD51-ICD bound to NR4A3 in the nucleus and regulated its transcriptional activity.

To identify the downstream effectors regulated by NR4A3, the NR4A3-FLAG vector was stably transfected in 293T cells. A ChIP-seq assay using anti-FLAG antibody was employed. We searched for PNI-related genes with specific ChIP-enriched peaks located in the promoter regions of the public database and found several potential genes reported previously, including NTRK1 [38], NTRK3 [39], and SEMA3E [40] (Figure 5D). As shown in Figure 5E, the direct binding of NR4A3 to the promoters of these genes was further validated by a ChIP-qPCR assay using primers designed against the binding site. As previously reported, NR4A3 could positively or negatively regulate downstream gene expression by recruiting different co-regulation factors, such as coactivators and corepressors (Figure 5F) [41]. Notably, NR4A3 overexpression suppressed the expression of these PNI-promoting genes. In contrast, CD51-ICD overexpression had an opposite effect on these genes. This phenomenon suggested that the interaction with CD51-ICD might reverse the transcriptional inhibition function of NR4A3. To test this hypothesis, we co-transfected the CD51-ICD vector and NR4A3 vector into the cells and found a further increase in the expression level of these three genes compared to the group transfected with only CD51-ICD (Figure 5G). These results confirmed our conjecture that CD51-ICD may act as a coactivator of NR4A3 and facilitate downstream gene expression.

Furthermore, we assessed the phenotype of NR4A3 affecting the neurotropism on cancer cell neurotropism at the cellular level. Both the co-culture migration assay (Figure 5H) and the time-lapse cell motility assay (Figure 5I) showed that overexpression of NR4A3 alone suppressed the neurotropism of cancer cells toward RSC96 cells, while cells transfected with the CD51-ICD vector exhibited the opposite phenomenon. However, simultaneous overexpression of NR4A3 and CD51-ICD within the same cells further enhanced its reactivity to RSC96 cells compared to the CD51-ICD group. Because TRKA encoded by NTRK1 and TRKC encoded by NTRK3 promote PNI through their interaction with nerve growth factor (NGF) and neurotrophin-3 (NT-3), we repeated the transwell assay and the time-lapse cell motility assay using the SN of RSC96 cells and obtained the same results (Figure S5A,B). Altogether, we proved that CD51-ICD interacted with NR4A3 to promote PNI in cancer cells, which might be explained by the coactivator effect of CD51-ICD on transcription regulation.

### 3.6. Pharmacological Inhibition of γ-Secretase Impedes PNI In Vitro and In Vivo

**Figure 6:**
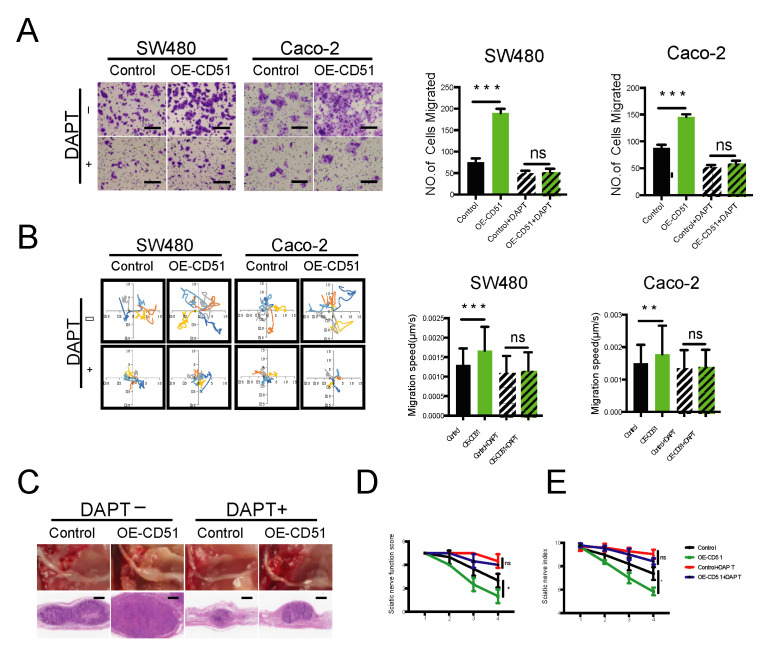
Pharmacological inhibition of γ-secretase impedes PNI in vitro and in vivo. (**A**) Statistics and representative images of the migration of SW480 and Caco-2 cells toward RSC96 cells with and without the γ-secretase inhibitor DAPT. *** *p* < 0.001, two-tailed Student’s *t*-test. Scale bars, 100 μm. (**B**) Wind rose plots and statistics of the migration speed of SW480 and Caco-2 cells stimulated by RSC96 cells with and without the γ-secretase inhibitor DAPT. Each curve represents a cell trajectory. ** *p* < 0.01, *** *p* < 0.001, two-tailed Student’s *t*-test. (**C**) In situ images of the sciatic nerve after injection for 4 weeks using SW480 cells transfected with empty vector or CD51 full-length vector. In addition, the corresponding H&E-stained images. Tumor-bearing mice were fed either with or without DAPT added to the diet. (**D**,**E**) Statistic of sciatic nerve function score and sciatic nerve index of mice after injection for 4 weeks using SW480 cells transfected with empty vector or CD51 full-length vector. Tumor-bearing mice were fed either with or without DAPT added to the diet. * *p* < 0.05, two-tailed Student’s *t*-test.

Based on our previous discovery that CD51 can be cleaved by γ-secretase to generate an ICD that promotes PNI, we employed the PNI models shown in Figure 2B,C to assess the effect of DAPT on cancer cell reactivity to RSC96 cell stimulation. Specifically, we co-cultured all cancer cells, including the control and OE-CD51 groups, with RSC96 cells in the presence or absence of DAPT. As shown in Figure 6A, CD51 overexpression led to a significant increase in cell migration induced by RSC96 cells, but this promoting effect was significantly inhibited after the addition of DAPT to the cells. In addition, the time-lapse cell motility assay further confirmed that the addition of DAPT to the culture system could abolish the increased cell movement caused by the overexpression of CD51 compared with the control group (Figure 6B). We repeated transwell assay and time-lapse cell motility assay using the SN of RSC96 cells and obtained the same results (Figure S6A,B). We proved that inhibition of γ-secretase could suppress the PNI of cancer cells at the cellular level.

To further assess the clinical relevance of our findings, we employed a mouse model to investigate the effect of γ-secretase inhibition on PNI in vivo. As shown in Figure 6C, tumors in the CD51 overexpression group were larger than those in the control group. However, the increase in tumor size was markedly reduced when DAPT was added to the diet. We quantified the damage to the sciatic nerve caused by cancer cells using the sciatic nerve function score and sciatic nerve index. Notably, the results obtained using these quantitative indicators were consistent with those obtained in the tumor size evaluation. Injection of CD51 overexpression cancer cells caused the most severe impairment among all groups, whereas the use of DAPT rescued the sciatic nerve from this damage (Figure 6D,E). In addition, to examine the effect of DAPT on organs in vivo, we extracted the livers and kidneys of mice treated with or without DAPT and found that DAPT did not cause significant damage to these organs (Figure S7A,B). In conclusion, our results confirmed that pharmacological inhibition of γ-secretase using DAPT demonstrated in vivo efficacy in improving the prognosis of PNI.

## 4. Discussion

PNI is defined as the invasion, surrounding, or pass-through of nerves by cancer cells. Specifically, tumor cells should be close to the nerve and surround at least 33% of the nerve periphery, or invade any of the three layers of the neurolemma structure [3]. PNI provides tumor cells with a metastasis channel independent of traditional blood and lymph vessels, thereby participating in tumor progression and recurrence [42]. The prognostic value of PNI has been well-established in various cancer types, including CRC [8,9,10,11]. However, a limited understanding of the mechanism of PNI in CRC restricts the need for effective intervention. Two previous studies [19,20] have reported that over-excretion of CD51 was correlated with an increased risk of PNI. On this basis, we applied the GSE103479 dataset in the GEO database and single-center data from the Sixth Affiliated Hospital of Sun Yat-Sen University to prove the upregulation of CD51 among CRC patients with PNI, in line with previous studies. In addition, we constructed two cancer cell lines with a stable knockdown of CD51 and identified the direct mediation relationship between CD51 and PNI using different models in vitro and in vivo. To the best of our knowledge, this is the first report that CD51 is directly responsible for regulating PNI.

Furthermore, we attempted to reveal the underlying molecular events mediating these processes. Integrins can be involved in regulating cellular fate such as survival, differentiation, and migration in several ways [43]. As a class of membrane proteins, integrins most commonly achieve their functions by binding to ligands and mediating the recruitment and assembly of downstream complexes, consisting of scaffolding and signaling proteins, in their cytoplasmic tails for signal transmission [44,45]. Over the decades, integrin trafficking into cells mediated by endocytosis has emerged as an alternative pathway to regulate its functions, especially in migrating cells [46]. This study found that the cellular localization of CD51 included both the membrane and nucleus. This suggested that in addition to functioning as a membrane protein, CD51 might also participate in some biological processes by entering cells to transmit signaling, similar to other integrins [47,48,49]. However, nuclear localization could only be detected using C-terminal antibodies. WB analysis of cytoplasmic and nuclear proteins further confirmed that no CD51 peptide in the nucleus could be recognized by N-terminal antibody. Therefore, we propose for the first time that CD51 could be processed to form a smaller fragment into cells.

Regulated intramembrane proteolysis (RIP) plays an important role in post-transcriptional regulation of some proteins for their further function. Four major types of intramembrane proteases have been identified, including S2P metalloprotease, SPP signal peptide peptidase, rhomboid protease, and γ-secretase. Among these, S2P metalloprotease cleaves type II transmembrane proteins, SPP signal peptide peptidase cleaves signal peptides, rhomboid protease cleaves proteins in the endoplasmic reticulum and Golgi, and only γ-secretase cleaves type I transmembrane proteins in the cell membrane transmembrane region [22]. Considering the existence of the CD51 C-terminal fragment in the cells, we focused on γ-secretase, which is responsible for transmembrane cleavage to generate ICD of proteins during RIP [50]. CD51 is a type I transmembrane protein that is the most common substrate for γ-secretase [22]; thus, it has the structural basis of cleavage. By sequence alignment, we found that there was a motif in the transmembrane region of CD51 that might be recognized and cleaved by γ-secretase. Further pharmacological inhibition assay confirmed that γ-secretase mediates the production of CD51-ICD. In addition, our results confirmed that CD51 exerts its PNI-promoting function through ICD, rather than the ECD segment. Collectively, the current study is the first to link γ-secretase-mediated endoproteolysis with the progression of PNI through CD51 as a mediator.

The release of soluble ICD is an important means of activating downstream molecules and mediating signal transmission such as the NOTCH pathway [51]. Numerous studies have elucidated the mechanism by which NOTCH generates ICD to regulate downstream gene expression via cleavage [52,53]. Generally, ICDs do not function directly as transcription factors but as transcription factor regulators by protein–protein interactions. For instance, Linda et al. reported that CD44-ICD acts as a co-transcription factor for RUNX2, thus contributing to cancer migration and progression [54]. Similar to previous studies, we found that CD51-ICD cannot bind to DNA directly as a transcription factor but acts as a regulator of the transcription factor NR4A3. Three genes, namely, *NTRK1* [38], *NTRK3* [39], and *SEMA3E* [40], reported to be associated with PNI, were recognized in our study as the downstream effectors regulated by NR4A3. Notably, the regulation of NR4A3 can be bidirectional, depending on their combination with coactivators or corepressors [41]. Our study proved that CD51-ICD could act as a coactivator to reverse the transcriptional suppression effect of NR4A3 on PNI-promoting genes and therefore be involved in the regulation of PNI.

Although several studies have pointed out the adverse effects of PNI on prognosis, effective treatments for PNI are still very limited. In our study, we reported the importance of γ-secretase-mediated cleavage events for CD51 in PNI promotion. Therefore, we wondered whether inhibiting this process could alleviate PNI in CRC. γ-Secretase inhibitors have been widely studied in the treatment of Alzheimer’s disease [23,55,56,57]. In addition, recent studies have expanded the application of γ-secretase inhibitors to cancers and developed multiple clinical trials [58,59,60]. However, no studies have reported a relationship between γ-secretase inhibition and PNI. Our study demonstrated that inhibiting γ-secretase can improve the treatment of PNI in CRC in preclinical models, and this effect is mediated at least in part by inhibition of CD51-ICD production, despite the known crosstalk between γ-secretase inhibition and the NOTCH pathway [24]. To our knowledge, this is the first study to bridge the gap between γ-secretase inhibition and PNI therapy. Of note, some previous studies have reported and documented the adverse effects caused by the administration of γ-secretase inhibitors [61] because of their wide range of substrates [62]. Although this may partially limit the clinical translation of the current study, the dilemma also encourages further efforts to explore new drugs with higher target specificity in the future [63].

## 5. Conclusions

In conclusion, our study provides evidence that CD51 participates in the promotion of PNI in CRC patients. γ-secretase plays a vital role in this process to generate CD51-ICD, which further interacts with the transcription factor NR4A3 to activate downstream signaling. More importantly, the pharmacological inhibition of γ-secretase suggests promising prospects for the treatment of PNI. Although there is a long path ahead, our findings provide a novel strategy, namely, targeting γ-secretase for the clinical treatment of PNI in CRC.

## Data Availability

All data were included in this article and the Supplementary File.

## Supplementary Materials

The following supporting information can be downloaded at: https://www.mdpi.com/article/10.3390/cancers15092623/s1, Figure S1: CD51 affects the neurotropism of CRC cells in vitro and in vivo; Figure S2: RSC96 induces an increase in CD51-ICD expression; Figure S3: the expression of ICD, ECD and OE-CD51 in colorectal cells and patient samples; Figure S4: Ectopic overexpression of CD51 ICD also promotes PNI in cancer cells; Figure S5: CD51-ICD regulates PNI by acting as a coactivator of transcription factor NR4A3; Figure S6: Pharmacological inhibition of γ-secretase impedes PNI in vitro and in vivo; Figure S7: DAPT did not cause significant damage to kidney and liver; Table S1: Primer sequences for quantitative polymerase chain reaction (qPCR); Table S2: Information for antibodies used in immunohistochemistry (IHC) and western blotting (WB); Table S3:The proteins interacting with CD51 intracellular domain (CD51-ICD) identified by mass spectrometry analysis.

## Abbreviations

APH-1	anterior pharynx defective-1
CD51	integrin alpha-V
CRC	colorectal cancer
ICD	intracellular domain
ECD	extracellular domain
ENS	enteric nervous system
NCT	nicastrin
NTRK1	neurotrophic receptor tyrosine kinase 1
NTRK3	neurotrophic receptor tyrosine kinase 3
PEN2	presenilin enhancer2
PNI	perineural invasion
PS	presenilin
SEMA3E	semaphorin 3E
