# Supplementary material for: CD51 Intracellular Domain Promotes Cancer Cell Neurotropism through Interacting with Transcription Factor NR4A3 in Colorectal Cancer
Source: Cancers (Basel). 2023 May 5;15(9):2623. doi: 10.3390/cancers15092623 (PMC10177513; doi:10.3390/cancers15092623)
Supplement: Supplementary file 1 [file cancers-15-02623-s001.zip › cancers-2257694-supplementary.pdf]

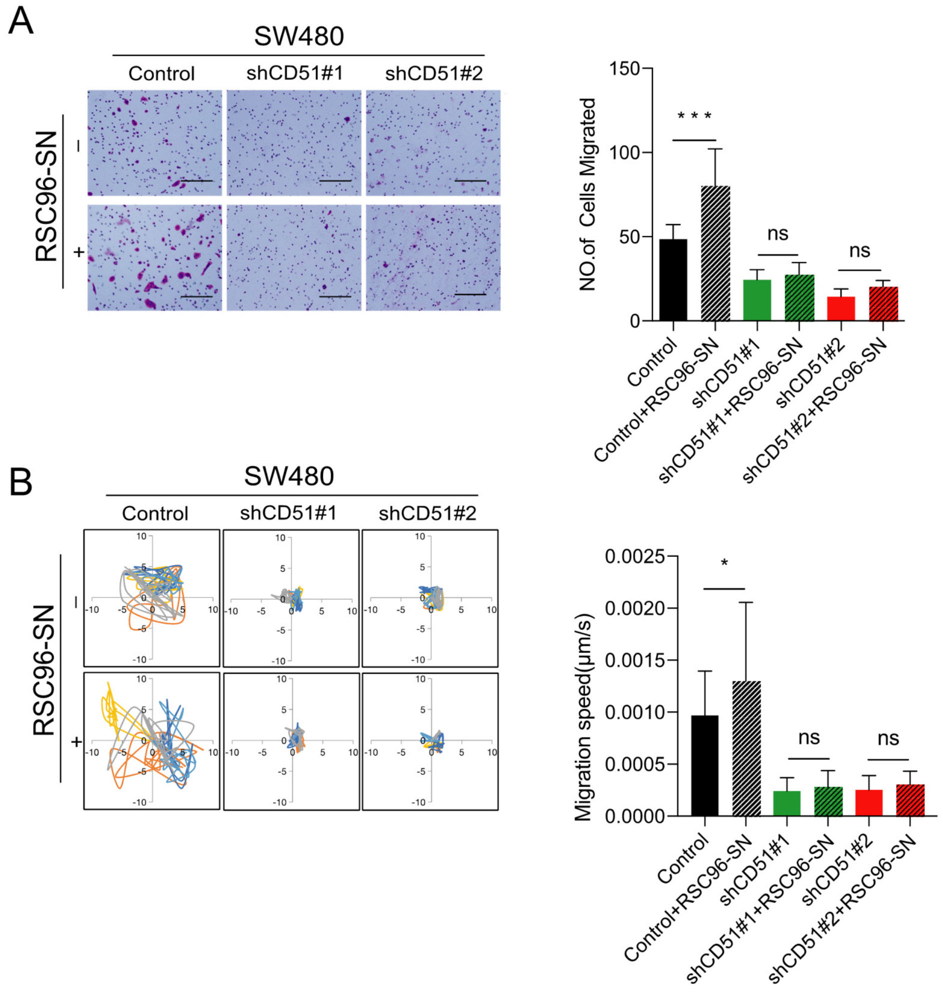

Figure S1. CD51 affects the neurotropism of CRC cells in vitro and in vivo.

(A) Statistics and representative images of migration of the shControl and shCD51 SW480 and Caco-2 cells. ***p<0.001, two-tailed Student's t-test. Scale bars, 100 μm.

(B) Wind-rose plots and statistics of migration speed of the shControl and shCD51 SW480 and Caco-2 cells. *p<0.05, two-tailed Student's t-test.

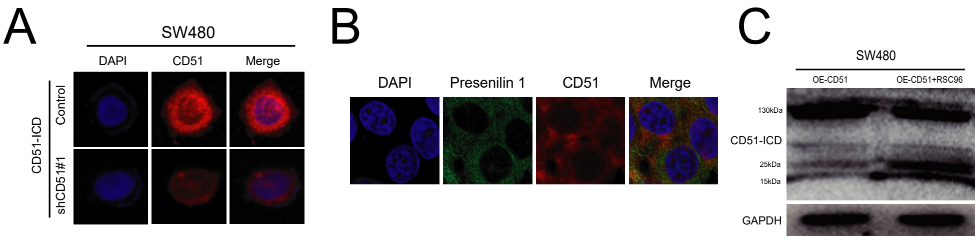

Figure S2. RSC96 induces an increase in CD51-ICD expression.

(A) Representative images of immunofluorescence staining using CD51-ICD antibody detection shControl and shCD51 cells.

(B) Representative images of immunofluorescence showed the detection of CD51 and presenilin 1.

(C) WB analysis showed an increase in protein expression at positions near 15 kDa after co-culturing tumor cells with RSC96 cells.

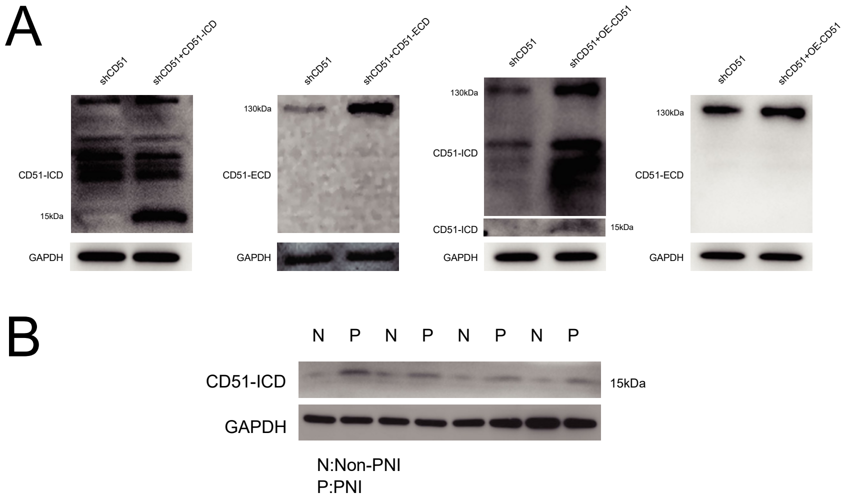

Figure S3. the expression of ICD, ECD and OE-CD51 in colorectal cells and patient samples

(A) WB analysis using the antibody which recognizes the CD51 intracellular and extracellular domain immunogen respectively to verify the ectopic overexpression in cancer cell lines.

(B) WB analysis showed an increase in CD51-ICD expression in patients with PNI.

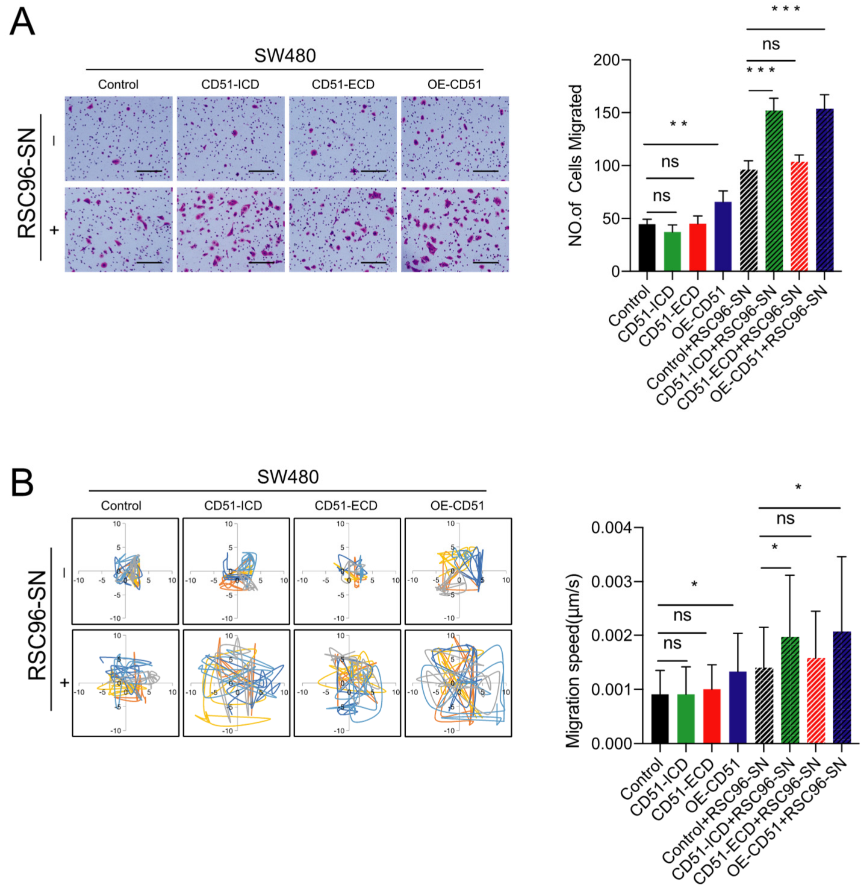

Figure S4. Ectopic overexpression of CD51 ICD also promotes PNI in cancer cells.

(A) Statistics and representative images of migration of the SW480 and Caco-2 cells transfected with empty vector, CD51 full-length vector, CD51-ICD vector and CD51-ECD vector. **$p<0.01$, ***$p<0.001$, two-tailed Student's t-test. Scale bars, 100 μm.

(B) Wind-rose plots and statistical analysis of the migration speeds of the SW480 and Caco-2 cells transfected with empty vector, CD51 full-length vector, CD51-ICD vector and CD51-ECD vector. *$p<0.05$，two-tailed Student's t-test.

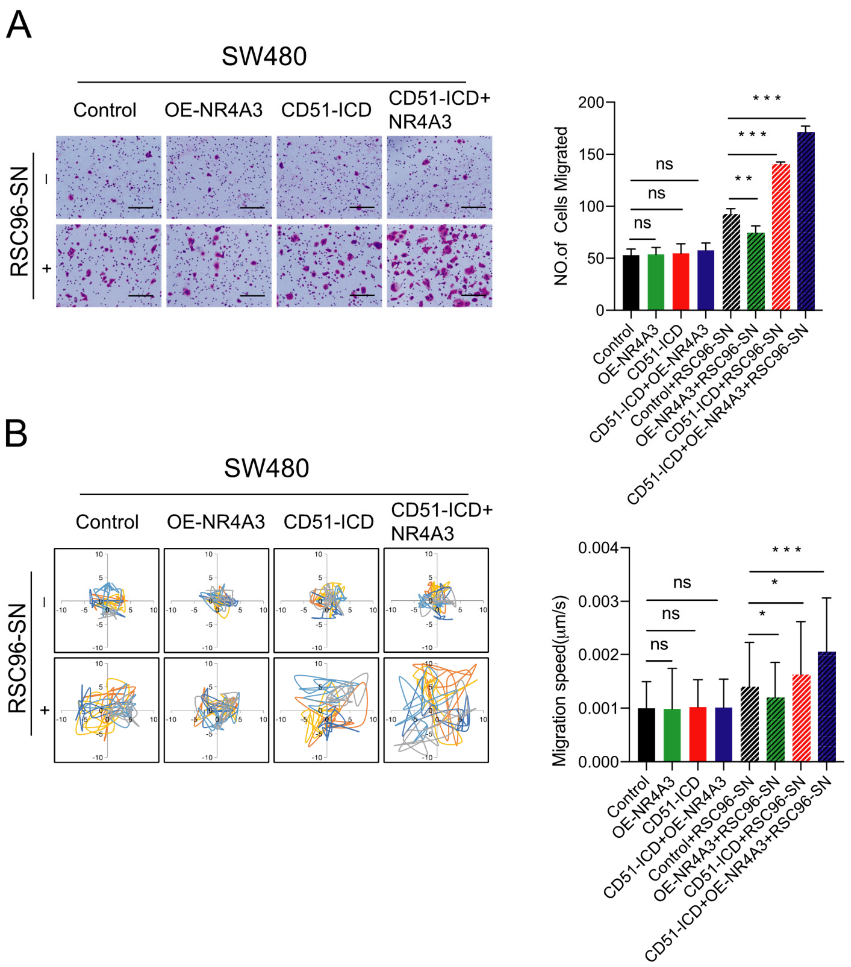

Figure S5. CD51-ICD regulates PNI by acting as a coactivator of transcription factor NR4A3.

(A) Statistics and representative images of the migration of cancer cells transfected with empty vector, NR4A3 vector, CD51-ICD vector, or both. **$p<0.01$，***$p<0.001$, two-tailed Student's t-test. Scale bars, 100 μm.

(B) Wind-rose plots and statistics of the migration speed of cancer cells transfected with empty vector, NR4A3 vector, CD51-ICD vector, or both. *$p<0.05$，***$p<0.001$, two-tailed Student's t-test.

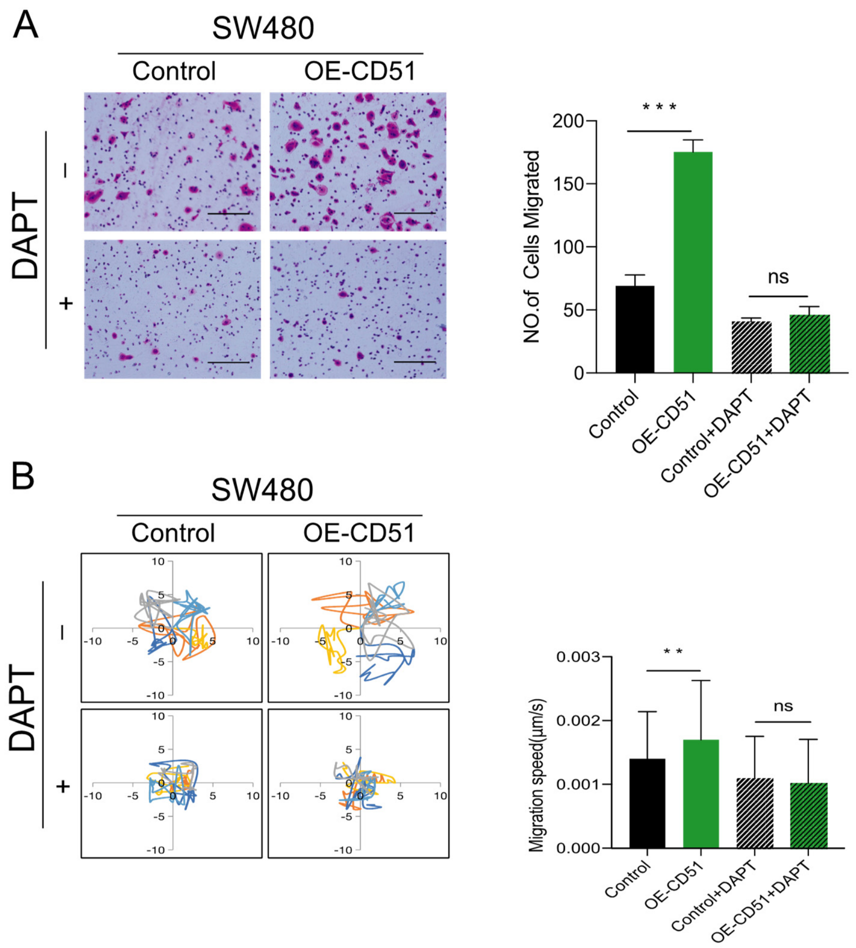

Figure S6. Pharmacological inhibition of γ-secretase impedes PNI in vitro and in vivo.

(A) Statistics and representative images of the migration of SW480 and Caco-2 cells toward RSC96-SN with and without the γ-secretase inhibitor DAPT. ***$p<0.001$, two-tailed Student's t-test. Scale bars, 100 μm.

(B) Wind-rose plots and statistics of the migration speed of SW480 and Caco-2 cells stimulated by RSC96-SN with and without the γ-secretase inhibitor DAPT. **$p<0.01$, two-tailed Student's t-test.

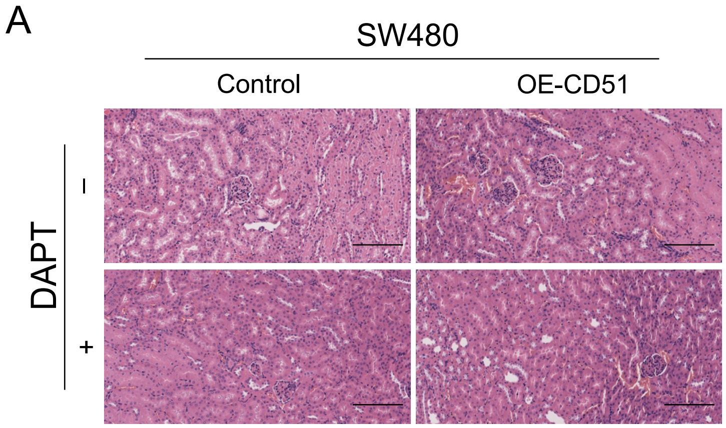

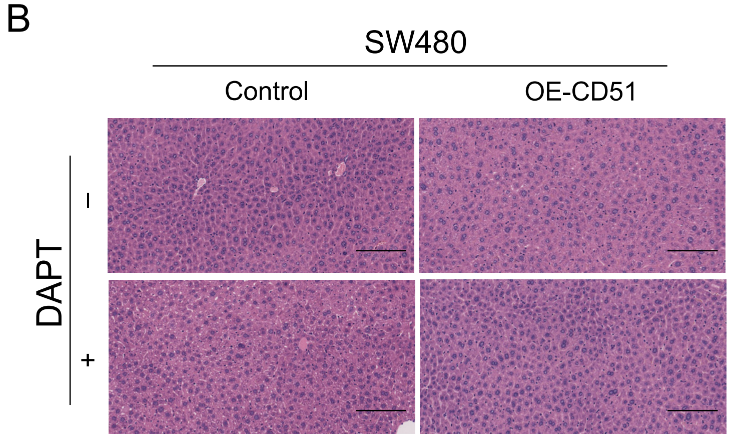

Figure S7. DAPT did not cause significant damage to kidney and liver.

(A) Representative images of kidneys. DAPT treatment did not show significant renal toxicity. Scale bars, 50 μm.

(B) Representative images of livers. DAPT treatment did not show significant liver toxicity. Scale bars, 50 μm.

Table S1. Primer sequences for quantitative polymerase chain reaction (qPCR).

Gene	Species	Type	Sequence
GAPDH	human	Forward primer (5'-3')	GGAGCGAGATCCCTCCAAAAT
		Reverse primer (5'-3')	GGCTGTTGTCATACTTCTCATGG
NTRK1	human	Forward primer (5'-3')	AACCTCACCATCGTGAAGAGT
		Reverse primer (5'-3')	TGAAGGAGAGATTCAGGCGAC
NTRK3	human	Forward primer (5'-3')	GCCAGTATCAACATCACGGAC
		Reverse primer (5'-3')	AGCCGGTTACTTGACAGGTTT
SEMA3E	human	Forward primer (5'-3')	GTTTGCTGGACTCTACAGTGAC
		Reverse primer (5'-3')	CTTTCAACAGACGCTCATCGT

Table S2. Information for antibodies used in immunohistochemistry (IHC) and western blotting (WB).

Name	Vendor	Catalog No.	Applications
S100	Proteintech	66616-1	IHC，WB
CD51-ECD	Proteintech	27096-1	IHC，WB
CD51-ICD	Merck millipore	AB1930	WB
GAPDH	Proteintech	60004-1	WB
NR4A3	Proteintech	55405-1	WB
HA	Cell Signaling Technology	3724S	WB

Table S3. The proteins interacting with CD51 intracellular domain (CD51-ICD) identified by mass spectrometry analysis.

Accession	Protein names	Gene names	MW [kDa]
Q13885	Tubulin beta-2A chain	TUBB2A	49.9
P68363	Tubulin alpha-1B chain	TUBA1B	50.1
P63267	Actin, gamma-enteric smooth muscle	ACTG2	41.9
P34931	Heat shock 70 kDa protein 1-like	HSPA1L	70.3
P11021	Endoplasmic reticulum chaperone BiP	HSPA5	72.3
Q15424	Scaffold attachment factor B1	SAFB	102.6
Q9UBS3	DnaJ homolog subfamily B member 9	DNAJB9	25.5
O75525	KH domain-containing, RNA-binding, signal transduction-associated protein 3	KHDRBS3	38.8
Q92598	Heat shock protein 105 kDa	HSPH1	96.8
Q15758	Neutral amino acid transporter B(0)	SLC1A5	56.6
P12272	Parathyroid hormone-related protein	PTHLH	20.2
Q12824	SWI/SNF-related matrix-associated actin-dependent regulator of chromatin subfamily B member 1	SMARCB1	44.1
Q96GM5	SWI/SNF-related matrix-associated actin-dependent regulator of chromatin subfamily D member 1	SMARCD1	58.2
P61964	WD repeat-containing protein 5	WDR5	36.6
P06576	ATP synthase subunit beta, mitochondrial	ATP5F1B	56.5
Q6NZY4	Zinc finger CCHC domain-containing protein 8	ZCCHC8	78.5
P51398	28S ribosomal protein S29, mitochondrial	DAP3	45.5
P21291	Cysteine and glycine-rich protein 1	CSRP1	20.6
P34096	Ribonuclease 4	RNASE4	16.8

Q01995	Transgelin	TAGLN	22.6
Q9BT67	NEDD4 family-interacting protein 1	NDFIP1	24.9
P08195	4F2 cell-surface antigen heavy chain	SLC3A2	68
Q14974	Importin subunit beta-1	KPNB1	97.1
Q9Y2R4	Probable ATP-dependent RNA helicase DDX52	DDX52	67.5
P02786	Transferrin receptor protein 1	TFRC	84.8
O95433	Activator of 90 kDa heat shock protein ATPase homolog 1	AHSA1	38.3
P49750	YLP motif-containing protein 1	YLPM1	241.5
Q6UWM7	Lactase-like protein	LCTL	65
P50990	T-complex protein 1 subunit theta	CCT8	59.6
P02647	Apolipoprotein A-I	APOA1	30.8
P11216	Glycogen phosphorylase, brain form	PYGB	96.6
Q03113	Guanine nucleotide-binding protein subunit alpha-12	GNA12	44.3
O14980	Exportin-1	XPO1	123.3
B5ME19	Eukaryotic translation initiation factor 3 subunit C-like protein	EIF3CL	105.4
P62136	Serine/threonine-protein phosphatase PP1-alpha catalytic subunit	PPP1CA	37.5
P13861	cAMP-dependent protein kinase type II-alpha regulatory subunit	PRKAR2A	45.5
Q3ZCQ8	Mitochondrial import inner membrane translocase subunit TIM50	TIMM50	39.6
P41252	Isoleucine--tRNA ligase, cytoplasmic	IARS1	144.4
P22102	Trifunctional purine biosynthetic protein adenosine-3	GART	107.7
O95487	Protein transport protein Sec24B	SEC24B	137.3
Q9BQE9	B-cell CLL/lymphoma 7 protein family member B	BCL7B	22.2
P06733	Alpha-enolase	ENO1	47.1
P29692	Elongation factor 1-delta	EEF1D	31.1
Q13200	26S proteasome non-ATPase regulatory subunit 2	PSMD2	100.1
Q12840	Kinesin heavy chain isoform 5A	KIF5A	117.3
Q96HU8	GTP-binding protein Di-Ras2	DIRAS2	22.5
P35241	Radixin	RDX	68.5
Q9BY07	Electrogenic sodium bicarbonate cotransporter 4	SLC4A5	126.2
Q12965	Unconventional myosin-Ie	MYO1E	127

Q16576	Histone-binding protein RBBP7	RBBP7	47.8
P61204	ADP-ribosylation factor 3	ARF3	20.6
P42704	Leucine-rich PPR motif-containing protein, mitochondrial	LRPPRC	157.8
O43175	D-3-phosphoglycerate dehydrogenase	PHGDH	56.6
O95639	Cleavage and polyadenylation specificity factor subunit 4	CPSF4	30.2
Q8IXW5	Putative RNA polymerase II subunit B1 CTD phosphatase RPAP2	RPAP2	69.5
P06730	Eukaryotic translation initiation factor 4E	EIF4E	25.1
Q9NRG0	Chromatin accessibility complex protein 1	CHRAC1	14.7
P82930	28S ribosomal protein S34, mitochondrial	MRPS34	25.6
Q9C0A0	Contactin-associated protein-like 4	CNTNAP4	145.2
Q96L58	Beta-1,3-galactosyltransferase 6	B3GALT6	37.1
P06756	Integrin alpha-V	ITGAV	116
P23526	Adenosylhomocysteinase	AHCY	47.7
P48200	Iron-responsive element-binding protein 2	IREB2	105
Q13242	Serine/arginine-rich splicing factor 9	SRSF9	25.5
P11908	Ribose-phosphate pyrophosphokinase 2	PRPS2	34.7
Q92567	Protein FAM168A	FAM168A	26.2
Q86VP6	Cullin-associated NEDD8-dissociated protein 1	CAND1	136.3
Q99943	1-acyl-sn-glycerol-3-phosphate acyltransferase alpha	AGPAT1	31.7
Q8N5H7	SH2 domain-containing protein 3C	SH2D3C	94.4
Q5XPI4	E3 ubiquitin-protein ligase RNF123	RNF123	148.4
Q92570	Nuclear receptor subfamily 4 group A member 3	NR4A3	68.2
Q06830	Peroxiredoxin-1	PRDX1	22.1
Q9NY59	Sphingomyelin phosphodiesterase 3	SMPD3	71
A0A1W2PPM1	Cytoplasmic polyadenylated homeobox-like	CPHXL	45.7
Q96I25	Splicing factor 45	RBM17	44.9
P02788	Lactotransferrin	LTF	78.1
P67936	Tropomyosin alpha-4 chain	TPM4	28.5
Q9UIG0	Tyrosine-protein kinase BAZ1B	BAZ1B	170.8
